# The loss‐of‐function variant in *MFSD3* could play a crucial role in the pathogenesis of dementia with Lewy bodies

**DOI:** 10.1002/alz.086694

**Published:** 2025-01-03

**Authors:** Tetsuaki Kimura, Mutsumi Suganuma, Kayoko Sawamura, Yuya Asanomi, Nobuyoshi Shimoda, Noboru Ogiso, Tohru Hosoyama, Shumpei Niida, Kouichi Ozaki, Daichi Shigemizu

**Affiliations:** ^1^ National Center for Geriatrics and Gerontology, Obu, Aichi Japan; ^2^ Core Facility Administration, Research Institute, National Center for Geriatrics and Gerontology, Aichi Japan; ^3^ RIKEN Center for Integrative Medical Sciences, Yokohama, Kanagawa Japan; ^4^ Hiroshima University Graduate School of Biomedical and Health Sciences, Hiroshima, Hiroshima Japan

## Abstract

**Background:**

Dementia with Lewy bodies (DLB) is the second most common form of degenerative dementia in older people. The clinical feature of DLB includes cognitive impairment, visual hallucinations, parkinsonism, and fluctuating attention. Three genes, *SNCA* (‐synuclein), *APOE* (apolipoprotein E), and *GBA* (glucosylceramidase), have been convincingly demonstrated to be associated with DLB. Our previous studies reported that a variant in *MFSD3* (rs143475431, c.888T>A:p.C296*) is associated with DLB in the Japanese population through whole‐genome sequencing and association studies. However, the precise mechanisms that this variant contributes to DLB development remains unclear.

**Method:**

We introduced the *MFSD3* p.C296* variant into a human neural stem cell line using the CRISPR/Cas9 system. Subsequently, the mutant cells were analyzed for cell proliferation rate using CyQUANT Cell Proliferation Assay Kit. The mutant cells were further induced to differentiate into neurons and astrocytes to assess their differentiation ability. Additionally, we generated the homologous *Mfsd3* knockout (KO) mice using the CRISPR/Cas9 system. The neurogenesis was evaluated by the number of doublecortin positive cells in the hippocampus. The behavior of *Mfsd3* KO and wild‐type mice was analyzed using IntelliCage.

**Result:**

We revealed that the *MFSD3* p.C296* variant was associated with decreased cell proliferation and reduced neurogenesis in the human neural stem cell line analyses. This decline in neurogenesis was also observed in the hippocampal dentate gyrus of *Mfsd3* KO mice, and the *Mfsd3* KO mice had smaller hippocampi compared to the wild‐type mice. Furthermore, *Mfsd3* KO mice exhibited a reduced level of curiosity about the new environment.

**Conclusion:**

Our results demonstrate that loss of function of *MFSD3* affects neurons and the brain, as indicated by our studies using human neural stem cell lines and *Mfsd3* KO mice. Further functional verification will contribute to elucidating the mechanism of DLB pathogenesis.

